# Supplementary material for: Artificial intelligence system reduces false-positive findings in the interpretation of breast ultrasound exams
Source: Nat Commun. 2021 Sep 24;12:5645. doi: 10.1038/s41467-021-26023-2 (PMC8463596; doi:10.1038/s41467-021-26023-2)
Supplement: Supplementary file 1 — Supplementary Information [file 41467_2021_26023_MOESM1_ESM.pdf]

Supplementary Information: Artificial Intelligence System Reduces False-Positive Findings in the Interpretation of Breast Ultrasound Exams

Yiqiu Shen[1,*], Farah E. Shamout[2,*], Jamie R. Oliver[3,*], Jan Witowski[3], Kawshik Kannan[4], Jungkyu Park[5], Nan Wu[1], Connor Huddleston[3], Stacey Wolfson[3], Alexandra Millet[3], Robin Ehrenpreis[3], Divya Awal[3], Cathy Tyma[3], Naziya Samreen[3], Yiming Gao[3], Chloe Chhor[3], Stacey Gandhi[3], Cindy Lee[3], Sheila Kumari-Subaiya[3], Cindy Leonard[3], Reyhan Mohammed[3], Christopher Moczulski[3], Jaime Altabet[3], James Babb[3], Alana Lewin[3], Beatriu Reig[3], Linda Moy[3,5], Laura Heacock[3], Krzysztof J. Geras[3,5,1,✉]

[1]Center for Data Science, New York University
[2]Engineering Division, NYU Abu Dhabi
[3]Department of Radiology, NYU Grossman School of Medicine
[4]Department of Computer Science, Courant Institute, New York University
[5]Vilcek Institute of Graduate Biomedical Sciences, NYU Grossman School of Medicine
[*]Equal contribution
✉k.j.geras@nyu.edu

Contents

1 Supplementary Tables **2**

2 Supplementary Figures **9**

3 Reader Study on the External Test Set **14**

1 Supplementary Tables

Supplementary Table 1 | Ablation study on the training dataset size. We reported the AUROC of the AI system with 95% confidence intervals on the internal test set ($n = 79,078$ breasts) when the AI system was trained using 1%, 10%, 50%, and 100% of data. More training data led to a better AUROC.

Training data	AUROC (95% CI)	No. of exams	No. of breasts
1%	0.887 (0.877, 0.891)	2,092	3,642
10%	0.939 (0.933, 0.945)	20,916	37,097
50%	0.969 (0.967, 0.973)	104,581	185,509
100%	0.976 (0.972, 0.980)	209,162	369,582

Supplementary Table 2 | Experience of readers who participated in the reader study. We summarized the experience of readers who participated in the reader study, in terms of the estimated number of breast ultrasound reads per year and the number of years of experience. All readers are attending radiologists who specialize in breast imaging.

Reader	Estimated reads per year	Years of experience
Reader 1	6500	6
Reader 2	2500	7
Reader 3	3000	4
Reader 4	750	32
Reader 5	1500	13
Reader 6	600	3
Reader 7	6000	35
Reader 8	6500	6
Reader 9	6000	7
Reader 10	3500	40

Supplementary Table 3 | Reader study performance. We reported the observed values and 95% confidence intervals of AUROC, AUPRC, specificity, sensitivity, biopsy rate, PPV, and NPV achieved by the AI system and radiologists on the reader study set (n = 1,024 breasts). We also showed the mean and standard deviation of radiologists' performance. We calculated the specificity and sensitivity of the AI system by dichotomizing its probabilistic predictions to match the average reader's sensitivity and specificity respectively. We similarly calculated the biopsy rate, PPV, and NPV of the AI system by matching the average reader's sensitivity.

Reader	AUROC	AUPRC	Specificity(%)	Sensitivity(%)	Biopsy rate(%)	PPV(%)	NPV(%)
R1	0.955<br>(0.935, 0.978)	0.612<br>(0.492, 0.688)	79.5<br>(75.7, 81.5)	97.3<br>(93.4, 100.0)	26.0<br>(24.5, 30.7)	26.7<br>(22.3, 32.5)	99.7<br>(99.5, 100.0)
R2	0.960<br>(0.946, 0.978)	0.616<br>(0.522, 0.689)	72.6<br>(70.9, 75.1)	98.6<br>(96.1, 100.0)	32.5<br>(30.3, 34.3)	21.6<br>(18.4, 26.3)	99.9<br>(99.6, 100.0)
R3	0.916<br>(0.866, 0.940)	0.550<br>(0.411, 0.640)	85.0<br>(82.0, 87.9)	84.9<br>(73.8, 91.8)	20.0<br>(17.5, 23.2)	30.2<br>(23.8, 36.3)	98.7<br>(97.8, 99.4)
R4	0.930<br>(0.906, 0.962)	0.596<br>(0.441, 0.696)	85.5<br>(84.0, 87.1)	90.4<br>(84.3, 95.1)	19.9<br>(17.8, 22.6)	32.4<br>(24.5, 38.5)	99.1<br>(98.5, 99.6)
R5	0.924<br>(0.900, 0.964)	0.695<br>(0.599, 0.777)	83.6<br>(81.1, 86.2)	90.4<br>(86.9, 96.7)	21.7<br>(18.7, 24.2)	29.7<br>(23.6, 36.8)	99.1<br>(98.6, 99.8)
R6	0.904<br>(0.874, 0.923)	0.498<br>(0.354, 0.598)	74.0<br>(70.5, 77.6)	89.0<br>(83.3, 93.4)	30.5<br>(26.3, 33.3)	20.8<br>(17.0, 26.3)	98.9<br>(98.3, 99.3)
R7	0.925<br>(0.909, 0.947)	0.447<br>(0.371, 0.535)	76.4<br>(73.8, 78.7)	90.4<br>(84.3, 96.7)	28.3<br>(25.9, 31.2)	22.8<br>(18.6, 27.4)	99.0<br>(98.3, 99.7)
R8	0.920<br>(0.879, 0.959)	0.624<br>(0.496, 0.765)	86.1<br>(83.4, 89.6)	87.7<br>(80.0, 92.4)	19.1<br>(15.1, 22.2)	32.7<br>(25.9, 38.8)	98.9<br>(97.8, 99.3)
R9	0.902<br>(0.870, 0.941)	0.533<br>(0.435, 0.609)	83.7<br>(81.2, 85.7)	86.3<br>(79.2, 91.6)	21.3<br>(18.9, 24.1)	28.9<br>(25.0, 34.8)	98.8<br>(97.9, 99.3)
R10	0.900<br>(0.869, 0.930)	0.484<br>(0.417, 0.566)	80.7<br>(77.8, 83.6)	86.3<br>(78.9, 91.1)	24.1<br>(22.2, 27.1)	25.5<br>(20.2, 33.8)	98.7<br>(97.6, 99.2)
Avg	0.924 ± 0.020<br>(0.905, 0.944)	0.565 ± 0.072<br>(0.465, 0.625)	80.7 ± 4.7<br>(78.9, 82.6)	90.1 ± 4.3<br>(86.4, 93.8)	24.3 ± 4.5<br>(22.0, 26.5)	27.1 ± 4.1<br>(22.9, 33.1)	99.1 ± 0.4<br>(98.4, 99.5)
AI	0.962<br>(0.943, 0.979)	0.752<br>(0.675, 0.849)	85.6<br>(83.9, 88.0)	94.5<br>(89.4, 100.0)	19.8<br>(17.9, 22.1)	32.5<br>(26.9, 39.2)	99.1<br>(98.2, 99.6)

Supplementary Table 4 | Subgroup analysis results: benign vs. malignant. We reported the values and 95% confidence intervals of AUROC, AUPRC, specificity, sensitivity, biopsy rate, PPV, and NPV achieved by the AI system and radiologists on the subgroup analysis. In this analysis, we included 574 exams (n = 608 breasts) from the reader study that yielded biopsy-confirmed benign or malignant findings. We also showed the mean and standard deviation of radiologists' performance. We calculated the specificity and sensitivity of the AI system by dichotomizing its probabilistic predictions to match the average reader's sensitivity and specificity respectively. We similarly calculated the biopsy rate, PPV, and NPV of the AI system by matching the average reader's sensitivity.

Reader	AUROC	AUPRC	Specificity(%)	Sensitivity(%)	Biopsy rate(%)	PPV(%)	NPV(%)
R1	0.932<br>(0.913, 0.957)	0.635<br>(0.570, 0.717)	67.9<br>(64.5, 71.9)	97.3<br>(91.5, 100.0)	40.0<br>(36.8, 42.4)	29.2<br>(23.1, 33.5)	99.5<br>(98.6, 100.0)
R2	0.937<br>(0.923, 0.967)	0.636<br>(0.593, 0.758)	57.4<br>(52.7, 62.5)	98.6<br>(95.9, 100.0)	49.3<br>(44.1, 53.6)	24.0<br>(20.3, 27.7)	99.7<br>(99.0, 100.0)
R3	0.889<br>(0.855, 0.929)	0.576<br>(0.517, 0.722)	76.6<br>(72.6, 82.5)	84.9<br>(79.5, 90.6)	30.8<br>(25.2, 33.9)	33.2<br>(27.0, 39.9)	97.4<br>(96.0, 98.7)
R4	0.908<br>(0.879, 0.944)	0.619<br>(0.523, 0.748)	76.6<br>(73.2, 80.7)	90.4<br>(87.1, 96.1)	31.4<br>(26.8, 35.0)	34.6<br>(28.7, 40.7)	98.3<br>(97.6, 99.3)
R5	0.907<br>(0.878, 0.951)	0.709<br>(0.646, 0.794)	72.9<br>(67.2, 77.9)	90.4<br>(87.0, 95.2)	34.7<br>(29.4, 40.5)	31.3<br>(27.7, 37.3)	98.2<br>(97.4, 99.3)
R6	0.866<br>(0.831, 0.920)	0.525<br>(0.450, 0.605)	59.4<br>(56.1, 64.3)	89.0<br>(82.8, 93.8)	46.4<br>(41.1, 49.3)	23.0<br>(20.2, 25.9)	97.5<br>(96.4, 98.8)
R7	0.890<br>(0.859, 0.915)	0.478<br>(0.439, 0.534)	64.9<br>(60.2, 68.3)	90.4<br>(84.3, 95.5)	41.8<br>(38.2, 46.2)	26.0<br>(22.3, 29.1)	98.0<br>(96.2, 99.1)
R8	0.896<br>(0.850, 0.949)	0.639<br>(0.535, 0.735)	77.6<br>(75.1, 81.4)	87.7<br>(79.5, 95.3)	30.3<br>(25.8, 33.1)	34.8<br>(29.1, 41.9)	97.9<br>(96.4, 99.3)
R9	0.870<br>(0.821, 0.922)	0.555<br>(0.458, 0.663)	75.0<br>(70.6, 79.6)	86.3<br>(77.4, 95.3)	32.4<br>(26.8, 36.0)	32.0<br>(26.7, 35.9)	97.6<br>(96.0, 99.3)
R10	0.868<br>(0.836, 0.925)	0.516<br>(0.433, 0.662)	69.9<br>(66.5, 73.9)	86.3<br>(81.2, 93.8)	36.8<br>(32.6, 40.3)	28.1<br>(22.2, 32.9)	97.4<br>(96.4, 99.0)
Avg	0.896 ± 0.024<br>(0.874, 0.929)	0.589 ± 0.067<br>(0.557, 0.671)	69.8 ± 6.9<br>(67.7, 73.6)	90.1 ± 4.3<br>(86.8, 93.8)	37.4 ± 6.4<br>(33.1, 39.8)	29.6 ± 4.0<br>(25.2, 33.6)	98.1 ± 0.8<br>(97.3, 99.0)
AI	0.941<br>(0.922, 0.968)	0.762<br>(0.695, 0.841)	78.3<br>(74.7, 81.0)	95.9<br>(90.1, 98.6)	29.9<br>(26.8, 33.1)	36.3<br>(30.8, 40.7)	98.4<br>(97.1, 99.5)

Supplementary Table 5 | Subgroup analysis results: cancer subtypes. We compared the number of correctly identified malignant lesions between the AI system and radiologists. In this analysis, we included 72 exams (73 breasts, 97 lesions) from the reader study with biopsy-confirmed malignant findings. We stratified the lesions by their cancer subtype, histological grade, and biomarker profile. For each stratification, we reported the total number of lesions (n), the number of lesions identified as malignant by the AI, and the number of lesions identified as malignant by radiologists. We dichotomized AI's probabilistic predictions by matching radiologists' average specificity in the reader study.

**Lesion characteristics**	n	**AI**	**radiologists (mean $\pm$ std)**
Cancer Subtype			
Invasive ductal carcinoma	75	72	70.7 ± 2
Invasive lobular carcinoma	9	9	8.2 ± 0.4
Other invasive carcinoma	8	8	5.9 ± 2.2
Ductal carcinoma in situ (DCIS)	5	4	4.1 ± 0.7
Histologic Grade (Invasive Cancers)			
Well differentiated	9	9	8.2 ± 0.4
Moderately differentiated	35	33	32.2 ± 1.2
Poorly differentiated	39	38	37.5 ± 1.5
Histologic Grade (DCIS)			
Well differentiated	0	-	-
Moderately differentiated	4	3	3.1 ± 0.7
Poorly differentiated	1	1	1 ± 0.0
Biomarkers of Invasive Cancers			
ER/PR-positive, HER2-negative	55	52	50.4 ± 1.9
HER2-positive	25	25	23.8 ± 1.2
ER/PR/HER2-negative	8	8	7.9 ± 0.3

Supplementary Table 6 | Performance of the hybrid models. We reported the values and 95% confidence intervals of AUROC, AUPRC, specificity, sensitivity, biopsy rate, PPV, and NPV achieved by the hybrid models (see Methods section 'Hybrid model') that combine the predictions of AI with each of the ten radiologists (R1-R10) on the reader study set (n = 1,024 breasts). The delta values show the difference (hybrid model-radiologist) and 95% confidence intervals in each metrics between each hybrid model and its respective reader. We calculated the specificity and sensitivity of each hybrid model by dichotomizing its probabilistic predictions to match its respective reader's sensitivity and specificity respectively. We similarly calculated the biopsy rate, PPV, and NPV of each hybrid model by matching the its respective reader's sensitivity.

Reader	AUROC	AUPRC	Specificity (%)	Sensitivity (%)	Biopsy rate (%)	PPV (%)	NPV (%)
R1	0.972	0.806	87.9	97.3	18.2	38.2	99.8
	(0.957, 0.991)	(0.713, 0.887)	(85.0, 89.2)	(93.4, 100.0)	(16.4, 22.2)	(30.4, 45.5)	(99.5, 100.0)
Δ	0.017	0.195	8.4	0.0	-7.8	11.5	0.0
	(0.012, 0.025)	(0.124, 0.245)	(6.5, 10.7)	(0.0, 0.0)	(-9.9, -6.1)	(8.1, 13.2)	(0.0, 0.1)
R2	0.973	0.812	80.1	100.0	25.5	27.6	99.9
	(0.964, 0.990)	(0.754, 0.889)	(78.6, 82.1)	(100.0, 100.0)	(22.9, 27.4)	(24.1, 33.2)	(99.6, 100.0)
Δ	0.013	0.196	7.6	1.4	-7.0	6.0	0.0
	(0.009, 0.018)	(0.153, 0.263)	(6.2, 8.9)	(0.0, 3.9)	(-8.3, -5.7)	(4.5, 7.0)	(0.0, 0.0)
R3	0.958	0.768	90.4	87.7	15.0	40.9	98.9
	(0.935, 0.975)	(0.646, 0.867)	(88.3, 92.0)	(80.3, 93.5)	(13.0, 17.3)	(32.9, 48.3)	(98.1, 99.4)
Δ	0.042	0.218	5.5	2.7	-5.0	10.7	0.2
	(0.021, 0.068)	(0.157, 0.291)	(4.0, 7.1)	(0.0, 6.6)	(-6.5, -3.7)	(7.6, 14.5)	(0.0, 0.5)
R4	0.966	0.792	91.7	93.2	14.2	45.5	99.2
	(0.953, 0.987)	(0.699, 0.871)	(90.4, 93.0)	(84.6, 98.4)	(12.3, 16.1)	(38.7, 52.7)	(98.1, 99.8)
Δ	0.036	0.195	6.2	2.7	-5.8	13.2	0.1
	(0.021, 0.053)	(0.138, 0.272)	(4.5, 7.9)	(0.0, 7.7)	(-7.3, -4.2)	(9.5, 16.4)	(-0.4, 0.5)
R5	0.962	0.804	90.2	90.4	15.5	41.5	99.2
	(0.947, 0.983)	(0.721, 0.886)	(89.1, 92.0)	(86.9, 96.7)	(13.3, 17.7)	(33.8, 47.5)	(98.7, 99.8)
Δ	0.037	0.109	6.6	0.0	-6.2	11.8	0.1
	(0.019, 0.047)	(0.062, 0.169)	(5.5, 9.3)	(0.0, 0.0)	(-8.6, -5.1)	(9.2, 15.3)	(0.0, 0.1)
R6	0.962	0.766	91.6	95.9	14.2	44.8	99.1
	(0.950, 0.985)	(0.673, 0.864)	(90.3, 93.3)	(91.8, 100.0)	(12.1, 16.3)	(37.9, 50.0)	(98.5, 99.8)
Δ	0.058	0.268	17.6	6.8	-16.3	24.0	0.2
	(0.038, 0.099)	(0.162, 0.377)	(15.6, 20.4)	(2.7, 13.1)	(-18.8, -14.1)	(19.6, 28.3)	(-0.2, 0.8)
R7	0.959	0.780	81.1	90.4	24.0	26.8	99.1
	(0.942, 0.980)	(0.708, 0.857)	(78.8, 82.8)	(84.3, 96.7)	(21.9, 26.7)	(22.4, 32.7)	(98.4, 99.7)
Δ	0.034	0.333	4.6	0.0	-4.3	4.1	0.1
	(0.023, 0.046)	(0.291, 0.440)	(3.8, 5.7)	(0.0, 0.0)	(-5.4, -3.6)	(3.4, 5.4)	(0.0, 0.1)
R8	0.956	0.787	89.2	89.0	16.4	38.7	99.1
	(0.931, 0.976)	(0.693, 0.870)	(87.2, 92.4)	(82.2, 93.7)	(12.5, 18.7)	(31.8, 46.0)	(98.1, 99.4)
Δ	0.036	0.163	3.0	1.4	-2.7	6.0	0.2
	(0.016, 0.052)	(0.089, 0.232)	(2.2, 4.2)	(0.0, 3.3)	(-3.8, -2.0)	(4.2, 8.9)	(0.0, 0.3)
R9	0.952	0.762	88.9	86.3	16.5	37.3	98.8
	(0.931, 0.972)	(0.673, 0.834)	(86.6, 90.7)	(79.2, 91.6)	(14.2, 18.9)	(31.5, 43.2)	(98.0, 99.3)
Δ	0.051	0.229	5.2	0.0	-4.8	8.4	0.1
	(0.030, 0.068)	(0.175, 0.298)	(4.3, 6.7)	(0.0, 0.0)	(-6.2, -4.0)	(6.4, 11.5)	(0.0, 0.1)
R10	0.950	0.763	89.3	87.7	16.1	38.2	98.8
	(0.928, 0.970)	(0.693, 0.832)	(87.9, 91.0)	(80.0, 92.8)	(14.1, 18.3)	(32.5, 48.1)	(97.7, 99.3)
Δ	0.050	0.278	8.6	1.4	-8.0	12.7	0.1
	(0.034, 0.063)	(0.208, 0.391)	(6.7, 11.3)	(0.0, 3.3)	(-10.5, -6.3)	(8.8, 15.6)	(-0.2, 0.3)

Supplementary Table 7 | Error breakdown by BI-RADS. We reported the number of false positive biopsies (FP) and false negatives diagnoses (FN) of ten radiologists (R1-R10) and the respective hybrid models on the reader study set (n = 1,024 breasts). We divided FP and FN according to the BI-RADS scores given by each radiologist. We dichotomized the probablistic predictions of each hybrid model to match its respective readers' sensitivity (sens) and specificity (spec). Overall, hybrid models were able to reduce the number of FP while yielding the same number or fewer FN than the respective readers.

	**Overall**		**BI-RADS 1/2**		**BI-RADS 3**		**BI-RADS 4A**		**BI-RADS 4B**		**BI-RADS 4C**		**BI-RADS 5**	
	FP	FN	FP	FN	FP	FN	FP	FN	FP	FN	FP	FN	FP	FN
R1	195	2	0	1	0	1	128	0	42	0	19	0	6	0
R1-hybrid (sens)	115	2	0	1	3	1	45	0	42	0	19	0	6	0
R1-hybrid (spec)	115	2	0	1	3	1	45	0	42	0	19	0	6	0
R2	260	1	0	0	0	1	133	0	74	0	44	0	9	0
R2-hybrid (sens)	188	1	2	0	2	1	57	0	74	0	44	0	9	0
R2-hybrid (spec)	226	0	2	0	2	0	95	0	74	0	44	0	9	0
R3	143	11	0	5	0	6	55	0	70	0	18	0	0	0
R3-hybrid (sens)	91	10	0	5	0	5	3	0	70	0	18	0	0	0
R3-hybrid (spec)	129	9	2	5	3	4	36	0	70	0	18	0	0	0
R4	138	7	0	5	0	2	74	0	46	0	13	0	5	0
R4-hybrid (sens)	79	7	1	4	3	1	11	2	46	0	13	0	5	0
R4-hybrid (spec)	100	5	2	4	3	1	31	0	46	0	13	0	5	0
R5	155	7	0	7	0	0	101	0	43	0	11	0	0	0
R5-hybrid (sens)	93	7	0	7	2	0	37	0	43	0	11	0	0	0
R5-hybrid (spec)	93	7	0	7	2	0	37	0	43	0	11	0	0	0
R6	244	8	0	4	0	4	34	0	175	0	29	0	6	0
R6-hybrid (sens)	80	7	0	2	0	3	0	0	45	2	29	0	6	0
R6-hybrid (spec)	231	3	0	1	1	2	20	0	175	0	29	0	6	0
R7	223	7	0	1	0	6	101	0	57	0	65	0	0	0
R7-hybrid (sens)	179	7	1	1	9	6	47	0	57	0	65	0	0	0
R7-hybrid (spec)	179	7	1	1	9	6	47	0	57	0	65	0	0	0
R8	132	9	0	6	0	3	68	0	50	0	12	0	2	0
R8-hybrid (sens)	103	8	0	6	4	2	35	0	50	0	12	0	2	0
R8-hybrid (spec)	103	8	0	6	4	2	35	0	50	0	12	0	2	0
R9	155	10	0	6	0	4	136	0	18	0	0	0	1	0
R9-hybrid (sens)	106	10	1	6	0	4	86	0	18	0	0	0	1	0
R9-hybrid (spec)	106	10	1	6	0	4	86	0	18	0	0	0	1	0
R10	182	10	0	6	0	4	110	0	50	0	20	0	2	0
R10-hybrid (sens)	100	10	0	6	2	3	26	1	50	0	20	0	2	0
R10-hybrid (spec)	113	9	0	6	2	3	39	0	50	0	20	0	2	0

Supplementary Table 8 | High-confidence triage analysis. We experimented with varying the operating point to improve the confidence of the AI system. A very low threshold results in high NPV and enables the AI to confidently identify negative cases. On the other hand, a very high threshold results in high PPV and enables the AI to confidently prioritize cases that are highly suspicious of malignancy. For either triage scenario, we reported the values and 95% confidence intervals of sensitivity, specificity, and NPV/PPV, along with number of breasts (n) associated with each metrics.

**Triage**	**Sensitivity (%)**	**Specificity (%)**	**Reliability of triage decision**
negative	98.63%<br>(95.35%, 100%)<br>$n = 73$	77.71%<br>(74.82%, 80.19%)<br>$n = 951$	99.86% (NPV)<br>(99.59%, 100%)<br>$n = 740$
positive	52.05%<br>(40.84%, 63.77%)<br>$n = 73$	99.26%<br>(98.73%, 99.67%)<br>$n = 951$	84.44% (PPV)<br>(72.97%, 93.76%)<br>$n = 44$

Supplementary Table 9 | Distribution of ultrasound devices. Breakdown of studies in the NYU Breast Ultrasound Dataset subsets by ultrasound machine models. There was no bias in terms of device preference when splitting the studies into training, validation and test sets.

Device	Training set	Validation set	Test set
Affiniti 70G	79080	13329	14715
S1000	40097	6684	9148
S3000	29676	4937	5785
S2000	24701	4054	5655
LOGIQ7	6316	1035	1647
Xario	6029	947	1541
iU22	4988	803	1696
LOGIQ9	3659	585	544
TUS-A300	3540	618	954
Accuvix V10	2478	389	788
Antares	2468	395	709
LOGIQ5	2232	471	832
Sequoia	1868	263	28
Accuvix V20	1851	311	680
LOGIQE9	152	19	26
HDI 5000	10	6	1
LOGIQS8	8	1	5
Aixplorer	4	1	0
LOGIQS7	4	2	1
UGEO H60	1	0	0

2 Supplementary Figures

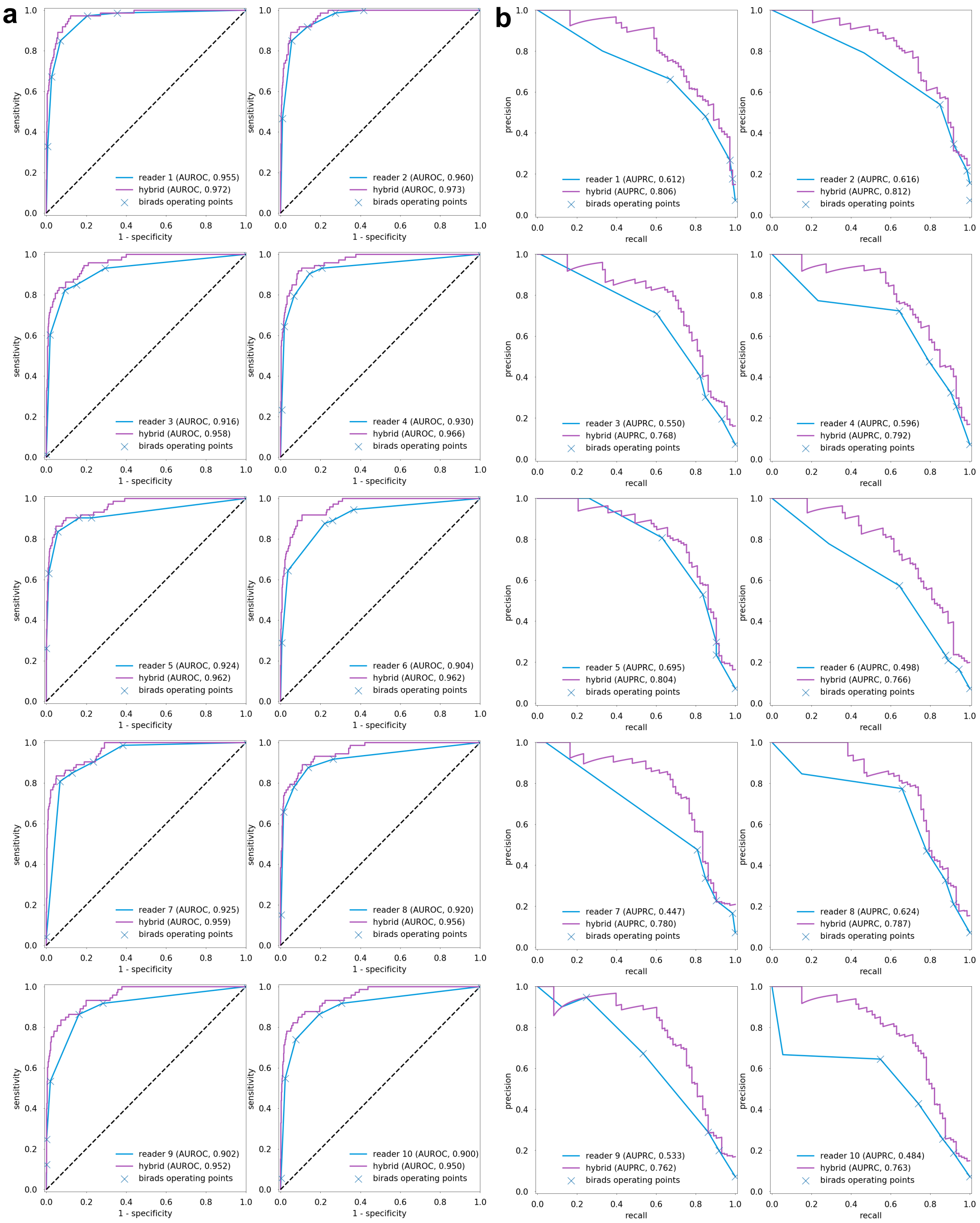

Supplementary Figure 1 | ROC and precision-recall curves for radiologists in the reader study. We visualized the ROC (**a**) and precision-recall curves (**b**) derived from the predictions made by ten radiologists and their corresponding hybrid models (see Methods section 'Hybrid model') in the reader study ($n = 1{,}024$ breasts). For each reader, we highlight the operating points which correspond to the performance this radiologist achieved when dichotomizing the radiologist's predictions using a threshold of BI-RADS categories (see Methods section 'Statistical analysis').

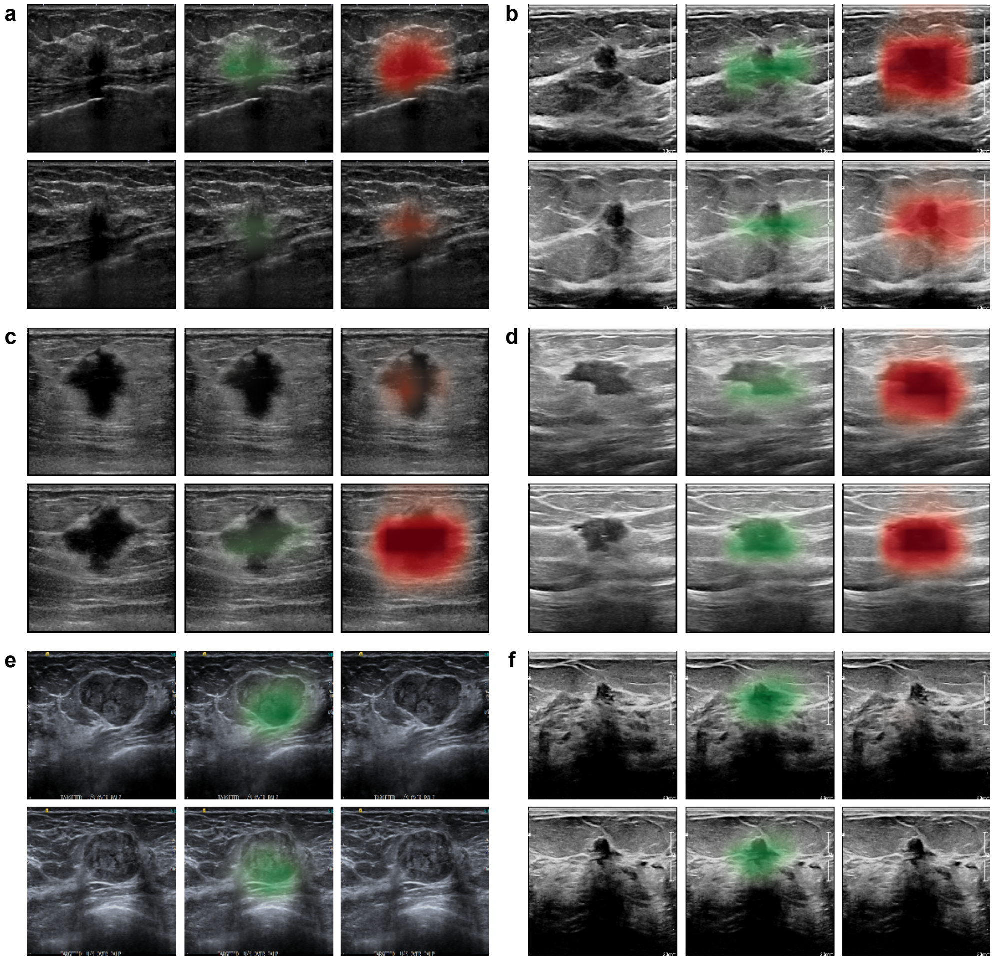

Supplementary Figure 2 | Additional saliency maps. We followed the same layout in Figure 3 and provided visualization of six cases from the internal test set. Exams **a-d** display lesions that were biopsied and found to be malignant (**a**: invasive mammary carcinoma, **b-d**: invasive ductal carcinoma). Exams **e** and **f** display lesions that were biopsied and found to be benign (fibroadenoma). The AI system correctly classified exams **a-d** as malignant and **e-f** as benign.

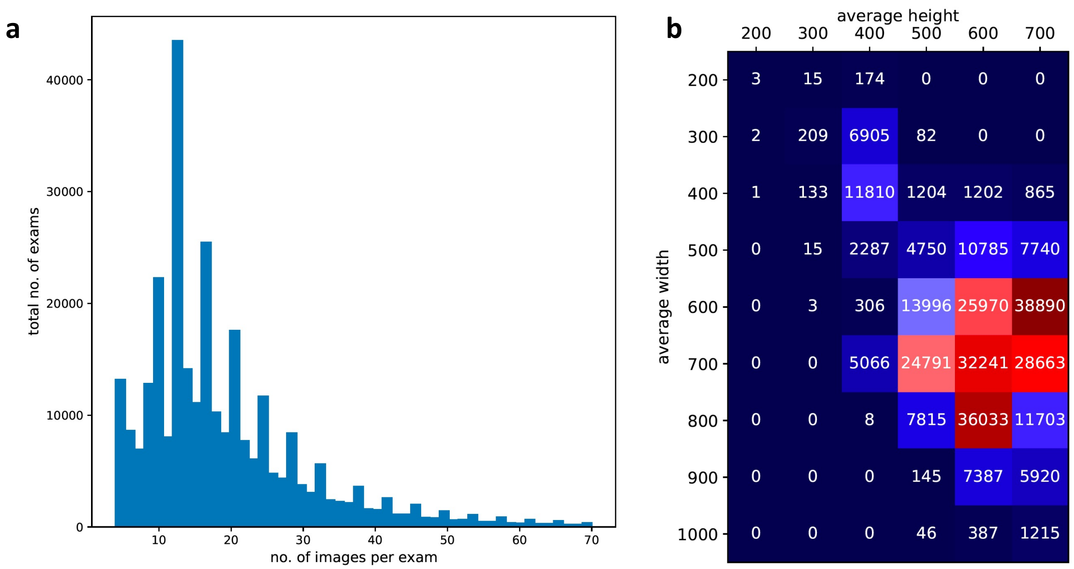

Supplementary Figure 3 | Number of images and resolution of images in the dataset. **a**, The distribution of the total number of images per exam. On average, each exam contains 18.8 US images. **b**, The distribution of the average size of the images in each exam. The x-axis represents the average image height per exam while the y-axis represents the average image width per exam (rounded to the nearest hundredth). The height and width are measured in number of pixels. The average resolution of images in this dataset is 665×603 pixels.

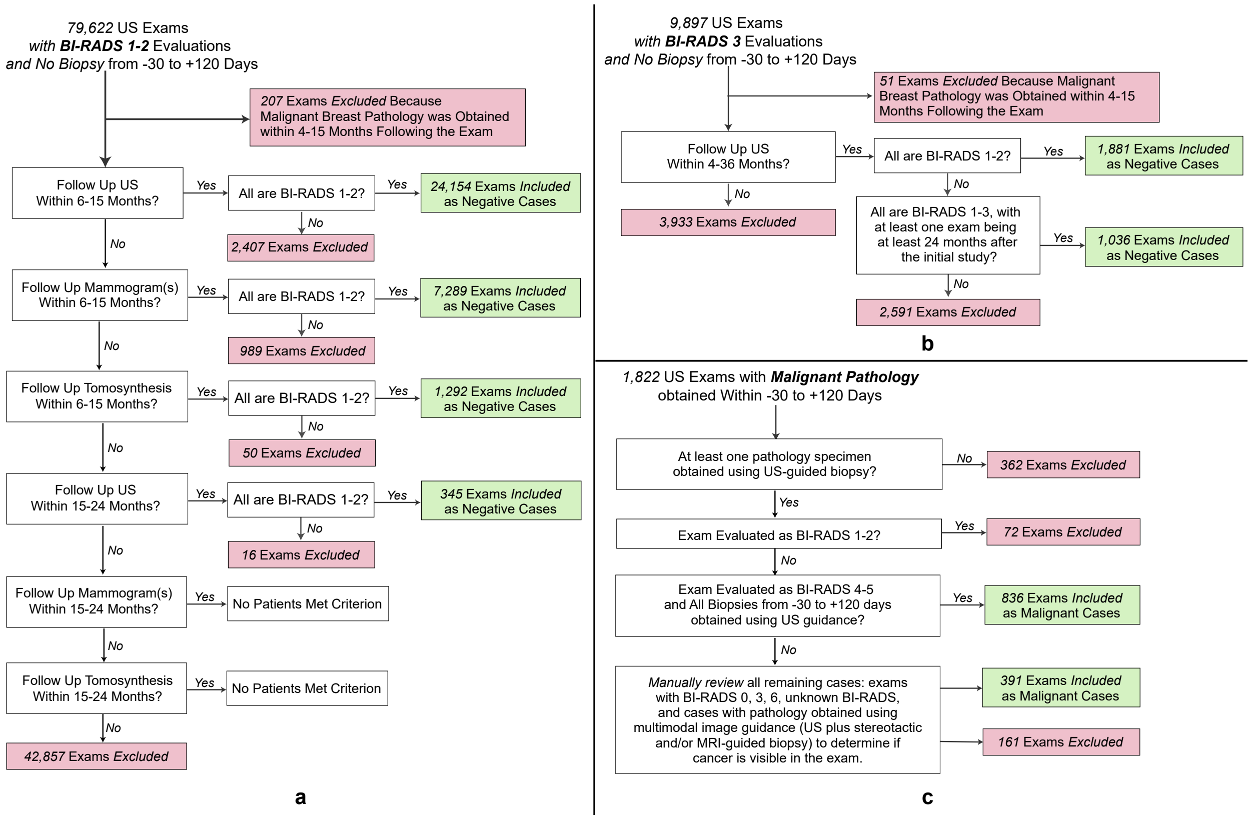

Supplementary Figure 4 | Filtering protocol applied on the internal test set. Cancer-negative exams were filtered to ensure that they are associated with a negative pathology report or have at least one cancer-negative follow-up. The specific workup for BI-RADS 1&2 and BI-RADS 3 exams were illustrated in **a** and **b** respectively. **c**, Exams with biopsy-proven cancers were filtered to ensure that cancers were visible on the US images.

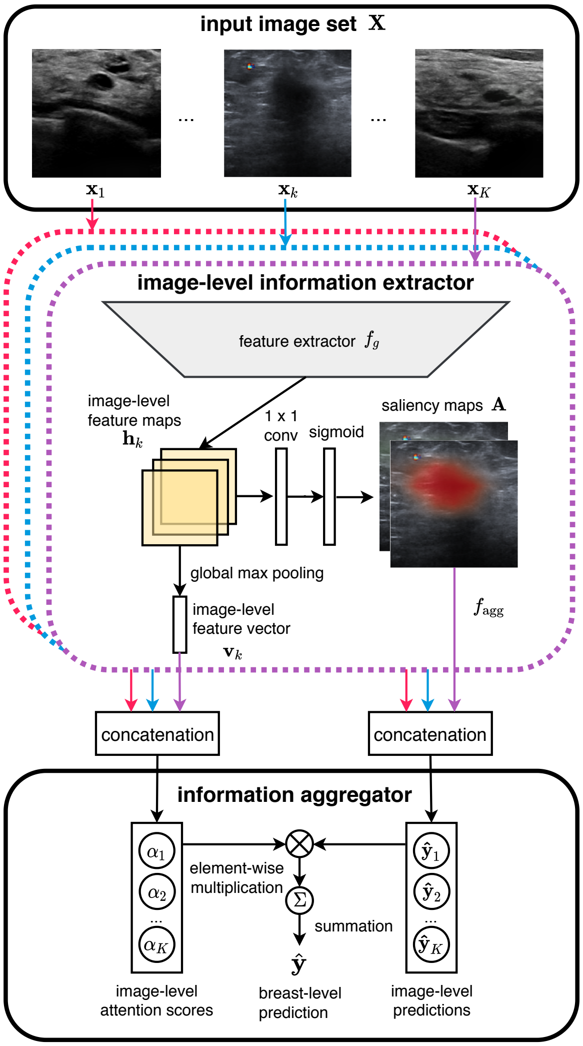

Supplementary Figure 5 | Overall structure of the deep neural network used in this study. The image-level information extractor first independently processes each ultrasound image $\mathbf{x}_k$ in the image set $\mathbf{X}$ and generates two saliency maps ($\mathbf{A}_k^b$, $\mathbf{A}_k^m$) that indicate the informative regions in the image. The network then calculates two attention scores (α_k^b, α_k^m) which indicate the importance of $\mathbf{x}_k$ for the diagnosis of benign and malignant lesions respectively. Lastly, the information aggregator then combines classification signals from all images and yields a breast-level prediction $\hat{\mathbf{y}}$.

Supplementary Table 10 | Reader study performance: the external test set We reported AUROC, AUPRC, specificity, sensitivity, biopsy rate, PPV, and NPV achieved by the AI system and radiologists on the external test set with 95% confidence intervals. In this analysis, we included all 780 images in the external test set. We also showed the mean and standard deviation of radiologists' performance. We calculated the specificity and sensitivity of the AI system by dichotomizing its probabilistic predictions to match the average reader's sensitivity and specificity respectively. We similarly calculated the biopsy rate, PPV, and NPV of the AI system by matching the average reader's sensitivity.

Reader	AUROC	AUPRC	Specificity(%)	Sensitivity(%)	Biopsy rate(%)	PPV(%)	NPV(%)
A	0.883<br>(0.855, 0.910)	0.706<br>(0.644, 0.772)	83.2<br>(80.2, 86.2)	85.7<br>(80.4, 90.0)	35.4<br>(32.4, 38.7)	65.2<br>(59.3, 71.9)	94.0<br>(91.7, 95.9)
B	0.893<br>(0.863, 0.925)	0.764<br>(0.713, 0.825)	79.1<br>(75.3, 82.4)	88.6<br>(83.9, 93.3)	39.1<br>(36.2, 42.7)	61.0<br>(55.3, 66.7)	94.9<br>(92.9, 97.1)
C	0.889<br>(0.862, 0.916)	0.746<br>(0.691, 0.804)	79.8<br>(76.5, 83.2)	88.1<br>(84.1, 92.2)	38.5<br>(35.1, 42.1)	61.7<br>(55.9, 67.2)	94.8<br>(92.9, 96.6)
Avg	0.888 ± 0.004<br>(0.855, 0.909)	0.739 ± 0.024<br>(0.632, 0.772)	80.7 ± 1.8<br>(78.8, 86.6)	87.5 ± 1.2<br>(81.2, 88.8)	37.6 ± 1.6<br>(32.4, 39.6)	62.6 ± 1.9<br>(58.1, 72.1)	94.6 ± 0.4<br>(92.3, 95.7)
AI	0.927<br>(0.907, 0.959)	0.858<br>(0.814, 0.897)	84.2<br>(82.6, 88.8)	90.5<br>(87.3, 94.4)	35.1<br>(32.2, 38.8)	67.2<br>(59.6, 74.5)	94.9<br>(92.9, 97.0)

3 Reader Study on the External Test Set

In order to compare the performance of the AI system with radiologists, we conducted a reader study on the external test set [1]. This dataset contains 780 ultrasound images and each image is associated with a binary label indicating the presence of any visible malignant lesions (see Methods section 'Breast Ultrasound Images Dataset'). Three board-certified breast radiologists rated each image according to the Breast Imaging Reporting and Data System (BI-RADS) [2]. No other information beyond ultrasound images was provided to the readers. Radiologist A has 4 years experience after completing fellowship in breast imaging. Radiologist B has 20 years experience after completing fellowship in breast imaging. Radiologist C has 11 years experience after completing fellowship in breast imaging.

For each reader, we computed a receiver operating characteristic (ROC) curve and a precision-recall curve by comparing their BI-RADS scores to the ground-truth outcomes (see Methods section 'Statistical analysis'). The three radiologists achieved an average AUROC of 0.888 (SD: 0.004, 95% CI: 0.855, 0.909) and an average AUPRC of 0.739 (SD: 0.024, 95% CI: 0.632, 0.772). Compared to the average radiologist in this study, the AI system achieved a higher AUROC of 0.927 (95% CI: 0.907, 0.959) with an AUROC improvement of 0.039 (95% CI: 0.017, 0.058, $P<0.001$). We summarized the performance of AI and all readers in Supplementary Table 10.

References

[1] Al-Dhabyani, W., Gomaa, M., Khaled, H. & Fahmy, A. Dataset of breast ultrasound images. *Data in Brief* **28**, 104863 (2020).

[2] Sickles, E. A. *et al.* ACR BI-RADS® atlas, breast imaging reporting and data system. *Reston, VA: American College of Radiology* 39–48 (2013).
