# Supplementary material for: Artificial intelligence system reduces false-positive findings in the interpretation of breast ultrasound exams
Source: Nat Commun. 2021 Sep 24;12:5645. doi: 10.1038/s41467-021-26023-2 (PMC8463596; doi:10.1038/s41467-021-26023-2)
Supplement: Supplementary file 2 — Reporting Summary [file 41467_2021_26023_MOESM2_ESM.pdf]

nature portfolio

Corresponding author(s): Krzysztof J. Geras

Last updated by author(s): Sep 1, 2021

Reporting Summary

Nature Portfolio wishes to improve the reproducibility of the work that we publish. This form provides structure for consistency and transparency in reporting. For further information on Nature Portfolio policies, see our Editorial Policies and the Editorial Policy Checklist.

Statistics

For all statistical analyses, confirm that the following items are present in the figure legend, table legend, main text, or Methods section.

n/a	Confirmed	
☐	☒	The exact sample size (n) for each experimental group/condition, given as a discrete number and unit of measurement
☐	☒	A statement on whether measurements were taken from distinct samples or whether the same sample was measured repeatedly
☐	☒	The statistical test(s) used AND whether they are one- or two-sided<br>*Only common tests should be described solely by name; describe more complex techniques in the Methods section.*
☐	☒	A description of all covariates tested
☐	☒	A description of any assumptions or corrections, such as tests of normality and adjustment for multiple comparisons
☐	☒	A full description of the statistical parameters including central tendency (e.g. means) or other basic estimates (e.g. regression coefficient) AND variation (e.g. standard deviation) or associated estimates of uncertainty (e.g. confidence intervals)
☐	☒	For null hypothesis testing, the test statistic (e.g. F, t, r) with confidence intervals, effect sizes, degrees of freedom and P value noted<br>*Give P values as exact values whenever suitable.*
☒	☐	For Bayesian analysis, information on the choice of priors and Markov chain Monte Carlo settings
☒	☐	For hierarchical and complex designs, identification of the appropriate level for tests and full reporting of outcomes
☒	☐	Estimates of effect sizes (e.g. Cohen's d, Pearson's r), indicating how they were calculated

Our web collection on statistics for biologists contains articles on many of the points above.

Software and code

Policy information about availability of computer code

Data collection	No software was used for data collection.
Data analysis	Image preprocessing was performed using Python (3.7) with following packages: OpenCV (3.4), pandas (0.24.1), Numpy (1.15.4), PIL (5.3.0), and Pydicom (2.2.0). Deep learning model were created using PyTorch (1.1.0) and Torchvision (0.2.2). Evaluation metrics were computed using Sklearn (0.19.1).

For manuscripts utilizing custom algorithms or software that are central to the research but not yet described in published literature, software must be made available to editors and reviewers. We strongly encourage code deposition in a community repository (e.g. GitHub). See the Nature Portfolio guidelines for submitting code & software for further information.

Data

Policy information about availability of data

All manuscripts must include a data availability statement. This statement should provide the following information, where applicable:

- Accession codes, unique identifiers, or web links for publicly available datasets
- A description of any restrictions on data availability
- For clinical datasets or third party data, please ensure that the statement adheres to our policy

The external test set (BUSI dataset) is available at https://scholar.cu.edu.eg/?q=afahmy/pages/dataset. The NYU Breast Ultrasound Dataset is not currently permitted for public release by the institutional review board of NYU Langone Health due to privacy concerns. We published the following report explaining how this dataset was collected to promote reproducibility: https://cs.nyu.edu/~kgeras/reports/ultrasound_datav1.0.pdf.

Field-specific reporting

Please select the one below that is the best fit for your research. If you are not sure, read the appropriate sections before making your selection.

- [x] Life sciences
- [ ] Behavioural & social sciences
- [ ] Ecological, evolutionary & environmental sciences

For a reference copy of the document with all sections, see nature.com/documents/nr-reporting-summary-flat.pdf

Life sciences study design

All studies must disclose on these points even when the disclosure is negative.

Sample size

The NYU Breast Ultrasound Dataset was collected from NYU Langone Health system (New York, USA) across 20 imaging sites. This dataset contains 288,767 exams (5,442,907 images) acquired from 143,203 patients imaged between January 2012 and September 2019. Patients were then randomly split among training (60%), validation (10%) and test (30%) sets. After splitting, each patient appeared in only one of the training, validation, and test sets. The training set consisted of 3,930,347 images within 209,162 exams collected from 101,493 patients. The validation set consisted of 653,924 images within 34,850 exams collected from 16,707 patients. The test set consisted of 858,636 images within 44,755 exams collected from 25,003 patients. The training set was used to optimize learnable parameters in the models. The validation set was used to tune the hyperparameters and select the best models. The test set was used to evaluate the performance of the models selected using the validation set. We applied additional filtering on the test set as described in the next section.

The Breast Ultrasound Images (BUSI) Dataset contains 780 images that were acquired from 600 female patients whose ages ranged between 25 and 75 years old. The BUSI dataset was only used for evaluation. The AI system was not trained on any image from this dataset.

Data exclusions

We applied a filtering criteria to obtain the NYU Breast Ultrasound Dataset. This entailed the exclusion of exams with invalid patient identifiers, exams collected before 2012, exams collected from patients younger than 16 years of age, duplicate images, exams from non-female patients, and invalid images based on the ImageType attribute, which consisted of non-ultrasound images such as reports or demographic data screenshots. We further excluded images that were collected during biopsy procedures based on the DICOM file attributes of the image metadata, images with missing metadata information relating to the type of procedure, images with more than 80% zero pixels, exams with multiple patient identifiers or study dates, exams with extreme number of images, and exams with missing image laterality.

To provide a clinically realistic evaluation of the AI system, we additionally refined the test set. First, we ensured that each non-biopsied exam was followed with a subsequent cancer-negative exam. Next, we refined exams with biopsy-proven benign findings to determine if the pathology results were deemed by the radiologist to be concordant or discordant with the imaging features of the breast lesion. Lastly, we ensured that exams with biopsy-proven cancers contained images of these cancers. We refer the readers to the Methods section for more details.

Replication

All attempts at replication were successful. All models were trained for several runs to ensure reproducibility. On the internal test set, the performance of the AI system was repeatedly evaluated on different patients cohorts stratified by age, breast density, and equipment manufacturers.

Randomization

All patients in the NYU Breast Ultrasound Dataset was randomly split into training (60%), validation (10%) and test (30%) sets. The data from a single patient could only appear in the training, validation, or test set. None of the images in the Breast Ultrasound Images Dataset was used for training or model selection.

Blinding

Blinding was not performed due to the retrospective nature of this study.

Reporting for specific materials, systems and methods

We require information from authors about some types of materials, experimental systems and methods used in many studies. Here, indicate whether each material, system or method listed is relevant to your study. If you are not sure if a list item applies to your research, read the appropriate section before selecting a response.

Materials & experimental systems

n/a	Involved in the study
x	Antibodies
x	Eukaryotic cell lines
x	Palaeontology and archaeology
x	Animals and other organisms
	x Human research participants
x	Clinical data
x	Dual use research of concern

Methods

n/a	Involved in the study
x	ChIP-seq
x	Flow cytometry
x	MRI-based neuroimaging

Human research participants

Policy information about studies involving human research participants

Population characteristics	In the NYU Breast Ultrasound Dataset, the mean age of the patients was 53.7. There were 26,843 breasts with benign findings and 5,593 breasts with malignant findings. Among all exams, the most frequent exam-level mammographic breast density was "breasts are heterogeneously dense", and the least frequent exam-level breast density was "breasts are entirely fatty". All ultrasound exams used in this study were from female patients.<br><br>The Breast Ultrasound Images Dataset contains 600 female patients whose ages ranged between 25 and 75 years old. No additional information was provided by the researchers who created this dataset.
Recruitment	Image data in the NYU Breast Ultrasound Dataset were collected from 143,203 patients, who met the inclusion criteria (see Methods section "Filtering of the dataset"), imaged at NYU Langone Health between January 2012 and September 2019.<br><br>Image data in the Breast Ultrasound Images Dataset were collected from 600 patients who imaged at Baheya Hospital for Early Detection and Treatment of Women's Cancer (Cairo, Egypt) in 2018.
Ethics oversight	This study was approved by the Institutional Review Board at NYU Langone Health (ID#i18-00712_CR3) and is compliant with the Health Insurance Portability and Accountability Act. Informed consent was waived since the study presents no more than minimal risk.

Note that full information on the approval of the study protocol must also be provided in the manuscript.
